# Evaluation of a quantitative measurement of suprapatellar effusion by ultrasonography and its association with symptoms of radiographic knee osteoarthritis: a cross-sectional observational study

**DOI:** 10.1186/s13075-016-1078-y

**Published:** 2016-08-04

**Authors:** Daisuke Chiba, Eiichi Tsuda, Shugo Maeda, Eiji Sasaki, Ippei Takahashi, Shigeyuki Nakaji, Yasuyuki Ishibashi

**Affiliations:** 1 Department of Orthopaedic Surgery, Hirosaki University Graduate School of Medicine, Hirosaki, Japan; 2 Department of Social Medicine, Hirosaki University Graduate School of Medicine, Hirosaki, Japan

**Keywords:** Knee osteoarthritis, Ultrasonography, Suprapatellar effusion, Knee symptoms

## Abstract

**Background:**

Quantitative measurement of knee joint effusion by ultrasonography has not been well established; however, a categorical measurement (e.g., a ≥4-mm-deep suprapatellar pouch) is recommended. Therefore, the current study aimed to elucidate the association between symptoms of knee osteoarthritis (OA) and the quantitative measurement of suprapatellar effusion by ultrasonography.

**Methods:**

One hundred twenty-seven volunteers participated (31 men and 96 women; mean age: 68.3 ± 9.8 years; body mass index: 23.2 ± 3.0 kg/m^2^). The Kellgren-Lawrence grades (KLGs) of both knees were assessed; all subjects had definitive osteoarthritic change (KLG ≥2) in both knee joints. Joint effusion was evaluated using an ultrasound probe, which was placed longitudinally on the suprapatellar pouch, and we determined the area (mm^2^) of the echo-free space. Then we summed the effusion area of both knees. All subjects answered the knee injury and osteoarthritis outcome scale (KOOS) questionnaire. Multiple linear regression analysis was conducted to elucidate the association between the summed value of the knee effusion area and the KOOS subscales, which were adjusted by age, sex, body mass index, and KLG.

**Results:**

Of 254 knees, 180 were KLG 2, 57 were KLG 3, and 17 were KLG 4. The multiple regression models showed that the quantitative knee effusion area significantly correlated with the following KOOS subscales: pain (*B* = −0.057; *β* = −0.253; *P* = 0.002), symptom (*B* = −0.053; *β* = −0.251; *P* = 0.002), sport and recreation (*B* = −0.069; *β* = −0.205; *P* = 0.007), and quality of life (*B* = −0.083; *β* = −0.276; *P* = 0.001).

**Conclusion:**

In this cross-sectional study, the quantitative measurement of suprapatellar effusion by ultrasonography was associated with symptoms of radiographic knee OA.

## Background

Knee osteoarthritis (OA) is a common joint disease that causes knee pain and stiffness, especially in the aging population. In our large-scale, domestic cohort, the prevalence rates of knee OA, which were estimated to be high in individuals aged ≥40 years, were 42.6 % in men and 62.4 % in women [1]. In this aging population, knee OA has a significant effect on general health. Knee pain secondary to OA impairs daily living activities [2] and leads to structural deterioration of the knee joint cartilage [3]. Thus, it is important to control and estimate the pain status of patients with knee OA.

Knee synovitis is accompanied by knee symptoms and cartilage destruction, and it induces synovial hypertrophy and the development of effusion in the joint cavity [4]. To determine the severity of synovitis, many researchers have used magnetic resonance imaging (MRI) (non-contrast or contrast-enhanced) [5, 6] and serum biomarkers [7–9]. Several previous studies have shown that it is possible to detect synovial hypertrophy and knee joint effusion using ultrasonography [10, 11]. However, most previous studies have only described the effect of knee effusion based on categorical evaluations such as the whole-organ magnetic resonance imaging score (WORMS) [12] using MRI or the European League Against Rheumatism recommendation [10] to determine whether the depth of the suprapatellar pouch is ≥4 mm or deeper on ultrasonography. Nevertheless, it is still unclear whether the quantitative evaluation of knee effusion has any effect in determining the patient’s pain status. Although there are numerous previous MRI studies on the diagnosis of knee synovitis, few studies have confirmed an association between knee synovitis and knee symptoms detected using ultrasonography. Therefore, the present study aimed to (1) establish a novel method for quantitatively measuring suprapatellar effusion on an ultrasonographic image by tracing the border of effusion and calculating its area, and (2) elucidate the association between the quantitative evaluation of suprapatellar effusion and pain status in Japanese patients.

## Methods

### The Iwaki Health Promotion Project

The Iwaki Health Promotion Project is a community-based program that promotes improvement of the average life span. This 10-year program was initiated in 2005. Approximately 1000 adults aged ≥20 years who live in the Iwaki area of Hirosaki City, Japan (the western Aomori prefecture) participate in this program annually. In addition to an orthopedic specialist, physicians, general surgeons, gynecologists, urologists, psychiatrists, dermatologists, and dentists are involved in this project. Our department collects biochemical and biomechanical data related to knee OA, including that of the spine, hip, and other joints.

### Subjects

Overall, 1167 participants underwent the health checkup in 2014. They answered questionnaires about their past and present medical history, lifestyle, occupation, family history, health-related quality of life, and disease-specific information such as knee symptoms. Of those who completed their questionnaire and underwent a radiographic examination, we randomly selected 500 participants to undergo a knee examination by ultrasonography because the number of eligible participants was limited. Finally, 127 volunteers who had radiographic OA of both knees were included in the current study. According to their responses, no participants had a history of hepatic or renal disease, malignancy, or rheumatoid arthritis. Subjects were also excluded if they had any history of severe knee trauma or knee surgery, or were taking any analgesic medications (e.g., non-steroidal anti-inflammatory drugs, acetaminophen, or tramadol) prescribed only by a medical institution. The Ethics Committee of the Hirosaki University Graduate School of Medicine approved the present study, and all subjects provided written informed consent before participation.

### Assessment of the radiographic data and knee symptoms

Radiographs of both knees were obtainerd in the standing position, and the knees were categorized based on the Kellgren-Lawrence grade (KLG) by one orthopedist (ES, who has 7 years of experience) [13]. In accordance with the KLGs, the severity of OA in knees with higher KLGs was defined on an individual basis. As aforementioned, all subjects had radiographic OA in both knees. Therefore, their knees were graded at least KLG 2 or more, and they had definitive marginal osteophytes or joint space narrowing. All participants completed the knee injury and osteoarthritis outcome scale (KOOS) questionnaire by themselves; staff members of our department assisted elderly subjects with this questionnaire. The KOOS is a 42-item, knee-specific, self-administered questionnaire with five subscales: pain, symptom, activities of daily living (ADL), sport and recreation (sports), and knee-related quality of life (QOL). All items were scored 0–4 and then summed. Then the raw scores were transformed to a 0–100 scale, in which 100 represents the best result and 0 represents the worst result. A separate score was calculated for each of the five subscales. The KOOS score is a sufficiently reliable, valid, and responsive tool for assessing pain or stiffness and other symptoms, including ADL, function in sport and recreation, and QOL associated with many types of knee disorders [14, 15].

### Ultrasonographic evaluation of suprapatellar effusion

**Fig. 1:**
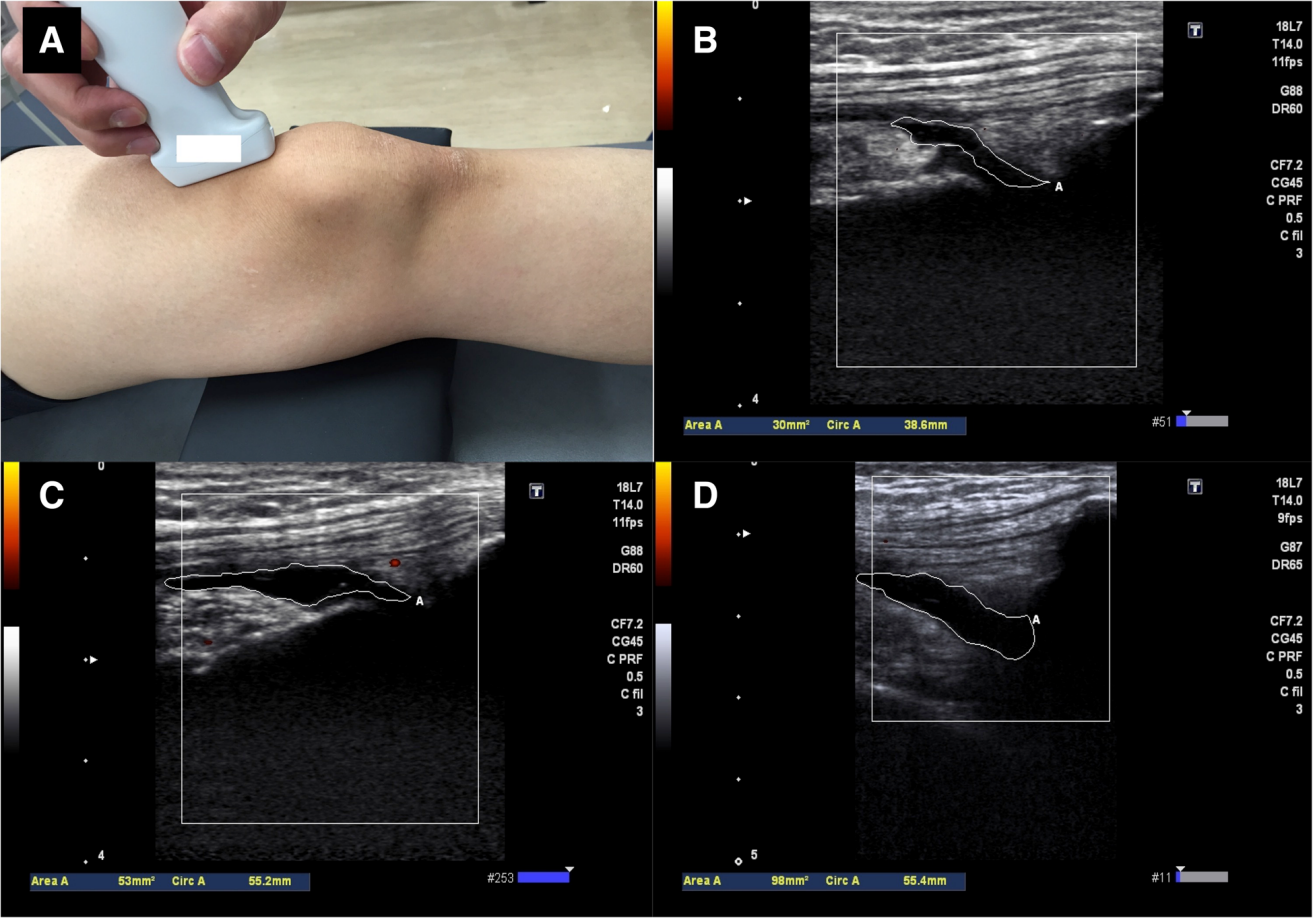
Representative images of the suprapatellar effusion area. **a** Ultrasound scan image of the suprapatellar effusion acquired by placing a linear probe longitudinally on the suprapatellar pouch. The effusion area was calculated by tracing the margin of the echo-free space that corresponds with the suprapatellar pouch. During the examination, the transducers should be placed on the subject as gently as possible to avoid distorting the acquired area. Representative images of the suprapatellar effusion area: 30 mm^2^ (**b**); 50 mm^2^ (**c**); and 100 mm^2^ (**d**)

To reduce bias, we performed ultrasonography after all participants had completed the questionnaire on knee pain. All ultrasound scans were evaluated by two examiners (DC, who has 5 years of experience in musculoskeletal ultrasonographic evaluation and SM, who has 8 years of experience). The subjects were examined lying in the supine position with both knees semi-flexed and their feet in the neutral position. The semi-flexed knee position was maintained by placing a pillow in both popliteal areas; in our preliminary data from 57 individuals, the knee flexion angle with this pillow was 20.0 ± 3.0°. Longitudinal ultrasound scans of the suprapatellar region were obtained; a linear transducer (12-MHz, Viamo^TM^; Toshiba Medical Systems Corp., Otawara, Japan) was gently placed over the same area at the center and proximal poles of the patella. On these images, echo-free space represented a suprapatellar effusion. While the margins of these echo-free spaces were traced, the area (mm^2^) of suprapatellar effusion was calculated automatically (Fig. 1). Finally, we summed the value of the effusion area in both knees to evaluate it effect on knee pain. During the examination, the transducers were placed as gently as possible, because pressure on the skin through the transducers should be avoided, as it can affect the acquired effusion area. For determining and calculating the knee effusion area in the suprapatellar pouch, the inter-rater reliability, intraclass/interclass correlation coefficient (ICC) (2,1) means the validation of evaluating effusion area, when two examiners test one subject, was 0.825 (95 % confidence interval (CI) 0.526, 0.935), and the intra-rater reliabilities, ICC (1,1) means the validation when one examiner test one subject, for examiners DC and SM were 0.991 (95 % CI 0.977, 0.997) and 0.988 (95 % CI 0.969, 0.995), respectively.

### Statistical analysis

The data were statistically analyzed using SPSS, version 22.0 J software (SPSS Inc., Chicago, IL, USA). First, we conducted analysis of variance (Tukey post-hoc test) to compare the mean values of the suprapatellar effusion area, age, body mass index (BMI), and KOOS subscales according to the KLG. Second, correlation between the suprapatellar effusion area and age, sex, BMI, KLG, or the KOOS subscales were determined by calculating the Spearman rank correlation coefficient. Third, to evaluate crude association between variables, single linear regression analysis was conducted with each KOOS subscale as the dependent variable and the suprapatellar effusion area as the independent variable. Finally, to evaluate the adjusted association between variables, multiple linear regression analysis was conducted with each KOOS subscale as the dependent variable and the suprapatellar effusion area as the independent variable, including age, sex, BMI, and KLG as the co-variables. A *P* value ≤0.05 was considered statistically significant.

## Results

### Comparison of the suprapatellar effusion area identified on ultrasonography, demographic data, and KOOS subscales among the KLGs

Of 254 knees (127 subjects), 180 (70.9 %) were KLG 2, 57 (22.4 %) were KLG 3, and 17 (6.7 %) were KLG 4. Among all the subjects, 81 (63.8 %) were KLG 2, 33 (26.0 %) were KLG 3, and 13 (10.2 %) were KLG 4. The mean age was significantly higher in subjects with KLGs 3 and 4 than in those with KLG 2. Additionally, the mean BMI was higher in subjects with KLG 4 than in those with KLGs 2 and 3. The mean scores of the KOOS subscales were significantly lower in subjects with KLGs 3 and 4 than in those with KLG 2 (Table 1).

**Table 1:** Comparison of the demographic values in subjects grouped by severity of knee osteoarthritis identified by radiography

	Total	KLG 2	KLG 3	KLG 4
	n = 127	n = 81	n = 33	n = 13
Age	68.3 ± 9.8	65.8 ± 9.8	71.5 ± 7.4^a^	75.4 ± 9.3^a^
Body mass index	23.2 ± 3.0	22.6 ± 2.7	23.6 ± 2.7	26.3 ± 3.7^ab^
Kellgren-Lawrence grade	4.7 ± 1.1	4.0 ± 0	5.5 ± 0.5^a^	7.3 ± 0.5^ab^
Effusion area	99.2 ± 89.7	73.9 ± 65.8	139.3 ± 94.8^a^	155.3 ± 139.8^a^
KOOS pain	83.6 ± 20.3	91.3 ± 12.2	73.1 ± 24.3^a^	62.8 ± 23.7^a^
KOOS symptom	84.5 ± 19.0	91.3 ± 13.3	75.9 ± 20.4^a^	64.3 ± 23.5^a^
KOOS ADL	88.8 ± 16.6	94.7 ± 10.0	80.4 ± 19.9^a^	74.0 ± 22.2^a^
KOOS sport	72.8 ± 30.1	85.1 ± 21.1	55.8 ± 29.3^a^	40.0 ± 36.1^a^
KOOS QOL	70.3 ± 27.1	82.4 ± 17.1	49.6 ± 28.3^a^	47.1 ± 29.6^a^

Statistical analysis by analysis of variance (Tukey post-hoc test); ^a^ *P* ≤ 0.01 compared to Kellgren-Lawrence grade (KLG) 2; ^b^ *P* ≤ 0.01 compared to KLG 3. The effusion area and KLG values are the sum of these values for both knees. *KOOS* knee injury and osteoarthritis outcome scale, *ADL* activities of daily living, *QOL* quality of life

In 254 knees, the mean values of the suprapatellar effusion area identified on ultrasonography were 39.2 ± 40.2 mm^2^ in those with KLG 2, 69.5 ± 59.3 mm^2^ in those with KLG 3, and 93.0 ± 91.3 mm^2^ in those with KLG 4. The suprapatellar effusion area increased with the progression of the KLG; the mean effusion area was significantly higher in those with KLGs 3 or 4 than in those with KLG 2 (Table 2).

**Table 2:** Comparison of the effusion area identified by ultrasonography according to the severity of knee osteoarthritis identified by radiography

Effusion area (mm^2^)	Total *N* = 254	KLG 2 *N* = 180	KLG 3 *N* = 57	KLG 4 *N* = 17
Average value	49.6 ± 52.4	39.2 ± 40.2	69.5 ± 59.3^*^	93.0 ± 91.3^*^
95 % CI	43.1, 56.1	33.3, 45.1	53.8, 85.3	46.0, 140.0

Statistical analysis was by analysis of variance (Tukey post-hoc test); ^*^ *P* ≤ 0.01 compared to Kellgren-Lawrence grade (KLG) 2. The effusion area of each knee joint was measured (n = 254 knees). *CI* confidence interval

### Correlation between the knee effusion and knee symptoms

**Fig. 2:**
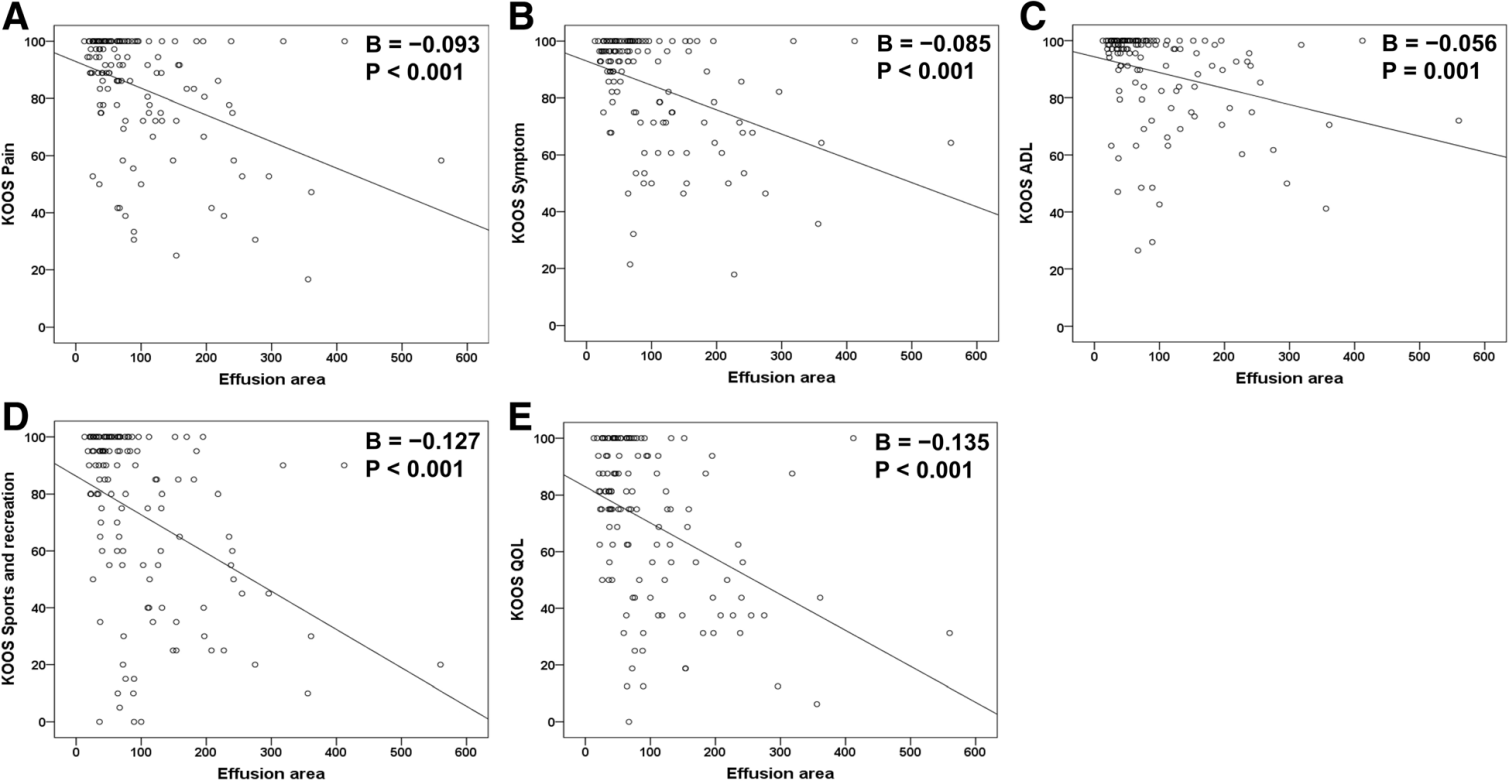
Scattergram of the suprapatellar effusion area and knee injury and osteoarthritis outcome scale (*KOOS*) subscales. The *y-axis* corresponds with each KOOS subscale, and the *x-axis* corresponds with the summed area (mm^2^) of suprapatellar effusion evaluated by ultrasonography (Fig. 1). The summed value of the effusion area was calculated by adding the value for the effusion areas of the right and left knees. **a** KOOS pain subscale; **b** KOOS symptom subscale; **c** KOOS activities of daily living (*ADL*) subscale; **d** KOOS sport and recreation subscale; **e** KOOS quality of life (*QOL*) subscale

According to Spearman correlation coefficient analysis, the suprapatellar effusion area assessed by ultrasonography had a significantly negative correlation with each KOOS subscale (Table 3). Results of single regression analysis also showed that the suprapatellar effusion area was inversely correlated with the following KOOS subscales: pain (*B* = −0.093; *β* = −0.413; *P* < 0.001), symptom (*B* = −0.085; *β* = −0.403; *P* < 0.001), ADL (*B* = −0.056; *β* = −0.301; *P* = 0.001), sports (*B* = −0.127; *β* = −0.419; *P* < 0.001), and QOL (*B* = −0.135; *β* = −0.400; *P* < 0.001) (Fig. 2). In the multiple regression model, the suprapatellar effusion area was consistently negatively correlated with the following KOOS subscales: pain (*B* = −0.057; *β* = −0.253; *P* = 0.002), symptom (*B* = −0.053; *β* = −0.251; *P* = 0.002), sports (*B* = −0.069; *β* = −0.205; *P* = 0.007), and QOL (*B* = −0.083; *β* = −0.276; *P* = 0.001). However, only the KOOS ADL subscale was insignificantly correlated with the suprapatellar effusion area (*B* = −0.024; *β* = −0.132; *P* = 0.109) when adjusted by age, sex, BMI, and KLG (Table 4).

**Table 3:** Correlation between the effusion area identified on ultrasonography, demographic data, and KOOS subscales

	Age	Sex	BMI	KLG	K-pain	K-sym	K-ADL	K-sports	K-QOL
Effusion area	0.187^*^	−0.158	0.142	0.422^**^	−0.382^**^	−0.370^**^	−0.373^**^	−0.441^**^	−0.433^**^
Age		0.076	0.135	0.353^**^	−0.143	−0.175^*^	−0.304^**^	−0.346^**^	−0.179^*^
Sex			−0.016	0.203^*^	−0.095	−0.160	−0.112	−0.039	−0.153
BMI				0.293^**^	−0.306^**^	−0.247^**^	−0.266^**^	−0.310^**^	−0.211^*^
KLG					−0.473^**^	−0.468^**^	−0.472^**^	−0.555^**^	−0.536^**^
K-pain						0.819^**^	0.843^**^	0.791^**^	0.877^**^
K-sym							0.754^**^	0.743^**^	0.789^**^
K-ADL								0.902^**^	0.798^**^
K-sports									0.755^**^

Statistical analysis was by Spearman rank correlation coefficient; ^*^ *P* ≤ 0.05; ^**^ *P* ≤ 0.01. The effusion area and KLG values are the sum of these values for both knees. *KLG* Kellgren-Lawrence grade, *K-pain* knee injury and osteoarthritis outcome scale (KOOS) pain subscale, *K-sym* KOOS symptom subscale, *K-ADL* KOOS activity of daily living subscale, *K-sports* KOOS sport and recreation subscale, *K-QOL* KOOS quality of life subscale

**Table 4:** Multivariate analysis of the factors associated with knee pain

Dependent variable	*R* ^2^ (adjusted)	Independent variables	*B*	*β*	*P* value	95 % CI
KOOS pain	0.375	Intercept	162.510	-	<0.001	132.506 to 192.514
		Age	−0.043	−0.021	0.780	−0.351 to 0.264
		Sex	−0.617	−0.013	0.859	−7.462 to 6.228
		BMI	−1.663	−0.247	0.001	−2.658 to −0.667
		KLG	−6.469	−0.358	<0.001	−9.565 to −3.374
		Effusion area	−0.057	−0.253	0.002	−0.092 to −0.022
KOOS symptom	0.344	Intercept	159.128	-	<0.001	130.282 to 187.974
		Age	−0.137	−0.070	0.361	−0.433 to 0.159
		Sex	2.378	−0.054	0.476	−8.959 to 4.203
		BMI	−1.226	−0.194	0.012	−2.184 to −0.269
		KLG	−5.788	−0.341	<0.001	−8.764 to −2.811
		Effusion area	−0.053	−0.251	0.002	−0.087 to −0.020
KOOS ADL	0.319	Intercept	165.423	-	<0.001	139.804 to 191.043
		Age	−0.312	−0.184	0.020	−0.575 to −0.050
		Sex	−0.189	−0.005	0.949	−6.034 to 5.656
		BMI	−1.333	−0.242	0.002	−2.184 to −0.483
		KLG	−4.561	−0.308	0.001	−7.204 to −1.918
		Effusion area	−0.024	−0.132	0.109	−0.054 to 0.006
KOOS sports	0.424	Intercept	212.530	-	<0.001	169.674 to 255.386
		Age	−0.510	−0.165	0.023	−0.950 to −0.070
		Sex	1.477	0.021	0.765	−8.300 to 11.255
		BMI	−2.241	−0.224	0.002	−3.664 to −0.819
		KLG	−10.292	−0.382	<0.001	−14.714 to −5.871
		Effusion area	−0.069	−0.205	0.007	−0.119 to −0.019
KOOS QOL	0.358	Intercept	169.047	-	<0.001	128.343 to 209.751
		Age	−0.133	−0.048	0.529	−0.551 to 0.284
		Sex	−7.883	−0.125	0.095	−17.170 to 1.403
		BMI	−1.102	−0.122	0.109	−2.453 to 0.249
		KLG	−8.894	−0.367	<0.001	−13.093 to −4.694
		Effusion area	−0.083	−0.276	0.001	−0.131 to −0.036

Statistical analysis was by multiple linear regression analysis using each knee injury and osteoarthritis outcome score (KOOS) subscale as the dependent variable; age, sex, body mass index (BMI), Kellgren-Lawrence grade (KLG), and the suprapatellar effusion area were used as the independent variables. The effusion area and KLG values are the sum of these values for both knees. *R* ^*2*^ coefficient of determination, *B* regression coefficients, *β* standardized regression coefficients, *sports* sport and recreation, *ADL* activities of daily living, *QOL* quality of life, *CI* confidence interval

## Discussion

To our knowledge, the current study is the first to report on the association between knee symptoms and the quantitative suprapatellar effusion area assessed using ultrasonography. The larger the quantitative effusion area, the lower the KOOS subscale scores. In this cross-sectional study, the suprapatellar effusion area significantly negatively affected knee symptoms in the population with radiographic evidence of knee OA, even after adjusting for age, sex, BMI, and severity of radiographic knee OA.

Several previous studies that used MRI and ultrasonography have reported that knee effusion worsens symptoms [16–19]. In a longitudinal study, Zhang et al. reported that knee effusion worsens patients’ pain status (Western Ontario and McMaster Universities osteoarthritis index pain scale) according to the whole organ magnetic resonance imaging score (WORMS) using MRI [16]. Another previous study demonstrated that in middle-aged women, knee effusion worsened pain and deteriorated their physical performance in activities such as timed walking and stair climbing [18]. Lo et al. performed a more detailed study in which effusion affected weight-bearing pain more than non-weight-bearing pain [19]. D’Agostino et al. performed ultrasonographic evaluations in a large symptomatic population and reported a high prevalence of suprapatellar effusion, which correlated with the development of sudden pain [10]. Similarly, Naredo et al. reported that the rest and motion pain scale scores were higher in those with suprapatellar effusion (≥4 mm deep) than in those with a lesser degree of effusion [20]. In a detailed review of knee synovitis, the most typical method used for determining the degree of synovitis is to evaluate the area of the joint cavity that is enlarged on imaging studies, because knee synovitis and effusion are associated with each other [4, 5]. This evidence supports the evaluation of knee effusion as an effective method for determining the pain status of an individual.

Conversely, recent reports have mentioned that knee effusion detected using ultrasonography was not correlated with patients’ pain status [21, 22]. Bevers et al. reported that there was no significant correlation between suprapatellar effusion and knee pain; they included subjects who were on analgesic medications [21], so the result of this study should be interpreted with caution, as certain nonsteroidal anti-inflammatory drugs compromise knee pain and synovitis [23]. Hall et al. reported that the association between suprapatellar effusion and the pain scale score was significant but weak; thus, they concluded that knee effusion is simply a phenomenon caused by the constructive degeneration of knee OA such as the decrease in lymph vessels of the synovium [22]. In the current study, we used the multiple regression model and included the severity of radiographic knee OA to adjust for the inter-relationship of constructive knee OA severity; even when adjusted, quantitative measurement of knee effusion had a significant effect on the pain status as assessed using the KOOS subscales patients with definitive radiographic evidence of knee OA. Although we agree that structural degeneration is one of the significant factors associated with the development of effusion in osteoarthritic knees, it is still difficult to determine which of the two, structural degradation or synovitis, is a more important factor that affects the development of effusion, especially in patients with radiographic knee OA. Further studies are needed to clarify the mechanism.

There are several limitations to the present study. First, this was a cross-sectional study; thus, future longitudinal studies are needed to clarify the association between knee symptoms and suprapatellar effusion. Second, we only examined the suprapatellar pouch. Thus, it is still unclear whether other quantitative measurements are associated with knee synovitis, such as synovial hypertrophy, and that of other sites with knee effusion, such as the medial or lateral recess. Additionally, we only examined one sagittal image of the suprapatellar pouch and only one image with the knee in the semi-flexed position. Third, the number of subjects with higher KLGs was small, because this was primarily a study of healthy individuals, as there were only a few subjects with severe OA. Although most subjects had any osteoarthritic knees, the present data are meaningful, because we surveyed these individuals in the pre-hospitalized state and all subjects on medications were excluded. Moreover, we originally performed a quantitative evaluation to estimate the suprapatellar effusion area, and this quantitative knee effusion value represented the pain status of the population with radiographic knee OA. There are recent reports that knee effusion as identified on MRI is more likely to destroy the joint surface and lead to the development of radiographic evidence of knee OA, as seen in longitudinal cohort studies [24–26]. Longitudinal data is little showing that knee effusion identified by ultrasonography causes cartilage destruction or structural degenaration. However, ultrasound evaluation of knee effusion has been directly correlated with a catabolic biomarker and the serum cartilage oligomeric matrix protein level, and inversely correlated with an anabolic biomarker, the N-terminal propeptide of type II collagen [27]. Further investigation is warranted to clarify the pathological correlation between knee effusion identified by ultrasonography and the degree of knee pain or structural damage in patients with knee OA.

## Conclusions

We quantitatively measured the sagittal area of knee effusion in the suprapatellar pouch by using ultrasonography and tracing the border of effusion on the ultrasound image, which is a novel method. An increase in the suprapatellar effusion area corresponded with worsened KOOS subscales in the population with radiographic evidence of knee OA. The current data support the finding that knee effusion can indicate the pain status of patients with knee OA.

## Abbreviations

ADL, activities of daily living; BMI, body mass index; CI, confidence interval; ICC, intraclass/interclass correlation coefficient; KLG, Kellgren-Lawrence grade; KOOS, knee injury and osteoarthritis outcome score; MRI, magnetic resonance imaging; OA, osteoarthritis; QOL, quality of life; sports, sport and recreation; WORMS, whole organ magnetic resonance imaging score
